# Effectiveness of Single-Dose Oral Pilocarpine Administration in Patients with Sjögren’s Syndrome

**DOI:** 10.3390/diagnostics14010091

**Published:** 2023-12-30

**Authors:** Aoi Komuro, Norihiko Yokoi, Chie Sotozono, Shigeru Kinoshita

**Affiliations:** 1 Department of Ophthalmology, Kyoto Prefectural University of Medicine, Kyoto 602-0841, Japan; akomuro@koto.kpu-m.ac.jp (A.K.); csotozon@koto.kpu-m.ac.jp (C.S.); 2 Department of Frontier Medical Science and Technology for Ophthalmology, Kyoto Prefectural University of Medicine, Kyoto 602-0841, Japan; shigeruk@koto.kpu-m.ac.jp

**Keywords:** Sjögren’s syndrome, oral pilocarpine, meniscometry, visual analogue scale, fluorescein breakup time

## Abstract

In this study, we evaluated the effectiveness of a single-dose oral pilocarpine administration on tear film (TF), as well as dry eye and dry mouth symptoms, in 53 eyes of 27 Sjögren syndrome (SS) patients who were experiencing dry mouth. To evaluate the changes in tear volume, a digital video-meniscometer was used to measure the radius of the lower central tear meniscus curvature (R, mm) of each eye at prior to the administration of 5 mg oral pilocarpine, and at 15 (R:(15)), 30 (R:(30)), and 60 (R:(60)) minutes after administration. The fluorescein breakup time (FBUT, seconds) and ocular and oral dryness symptoms were evaluated before and at 60 min after administration using a visual analogue scale (VAS, mm). A significant increase in R was observed at 15 and 30 min after administration compared to that at prior to administration. FBUT showed significant improvement at 60 min after administration, and the VAS score for ocular and oral dryness symptoms was found to have decreased significantly at 60 min after administration. A single-dose administration of 5 mg oral pilocarpine had a beneficial effect on TF, as well as on ocular and oral dryness symptoms, in patients with SS.

## 1. Introduction

Sjögren’s syndrome (SS) is an autoimmune disease characterized by the involvement of exocrine glands, such as the salivary and lacrimal glands, that cause dry eye and dry mouth [1]. SS is classified as primary (PSS) when the clinical manifestations occur alone, or as secondary (SSS) when associated with another autoimmune disease, usually a connective tissue disease [1,2]. Although the pathogenesis of SS is still unknown, the involvement of Th17 cells has received increasing attention in recent years [3,4].

Tear secretion from the salivary and lacrimal glands is reportedly mediated by the M3 muscarinic acetylcholine receptor (M3R) [5,6], and rabbit lacrimal gland protein secretion is also mediated by cholinergic stimulation of the M3R [7]. Moreover, autoantibodies against M3R have been found to suppress salivary secretion [5], and it has been reported that anti-M3R antibodies may be involved in the pathogenesis of dry eye in SS cases [8,9,10].

Pilocarpine hydrochloride is a plant alkaloid and an M3R agonist derived from the leaves of both Pilocarpus jaborandi and Pilocarpus microphyllus in South America, and it has been reported that oral pilocarpine increases salivary flow in patients with SS [5,11,12,13]. In Japan, oral pilocarpine is approved for administration by the national health insurance program as a treatment for dry mouth associated with radiotherapy of the head and neck region, as well as dry mouth in SS patients. Oral pilocarpine also acts on M3Rs in the lacrimal gland and promotes tear secretion [14]. It has been reported that a 12-week treatment with pilocarpine hydrochloride improves oral and ocular dryness [12], increases conjunctival goblet cell density and improves tear-film breakup time (BUT) [15], significantly increases tear secretion in healthy adults at 2 h after oral administration [16], significantly improves ocular dryness symptoms compared to patients treated with artificial tears and those with inferior punctal occlusion [17], and improves tear meniscus height and corneoconjunctival epithelial damage [18]. Similarly, cevimeline hydrochloride, another acetylcholine M3R agonist, reportedly improves subjective symptoms [19,20], Schirmer’s levels and subjective symptoms at 12 weeks of treatment [21], tear dynamics, and corneoconjunctival epithelial damage at 1 month of treatment [22]. However, there are no published reports on the evaluation of tear dynamics immediately after oral administration in patients with SS. In addition, it is unclear as to whether or not those prolonged BUTs and increased Schirmer values occur immediately after a single-dose administration of oral pilocarpine hydrochloride.

Reflective meniscometry is a method used to optically measure the radius of the lower central tear meniscus curvature (R, mm) [23,24]. In a previous study, we found a primary correlation between R and aqueous tear volume on the ocular surface, and R can be an indicator to monitor changes in tear volume on the ocular surface [24]. Moreover, in previous studies, we used meniscometry to measure the changes in R over time in normal human eyes and aqueous deficient dry eye after the instillation of ophthalmic solutions [23,25,26]. In this present study, we investigated the effects of a single-dose administration of oral pilocarpine hydrochloride on lacrimal function using meniscometry and analyzed subjective symptoms in patients with SS.

## 2. Materials and Methods

### 2.1. Subjects

This study involved 53 eyes of 27 SS patients (1 male and 26 females; mean age: 52.9 ± 17.5 (mean ± standard deviation (SD)) years, range: 33 to 87 years) experiencing dry mouth who were seen at the Dry Eye Outpatient Clinic at the Kyoto Prefectural University of Medicine Hospital, Kyoto, Japan. In all subjects, SS was diagnosed based on Fox’s criteria; i.e., (1) objective evidence of keratoconjunctivis sicca, as documented by rose Bengal or fluorescein dye staining, (2) objective evidence of diminished salivary gland flow, (3) minor salivary gland biopsy obtained through normal mucosa with the specimen containing at least 4 evaluable salivary gland lobules and with an average of at least 2 foci/4 mm^2^, and (4) evidence of a systemic autoimmune process, as manifested by the presence of autoantibodies such as rheumatoid factor, anti SS-A antibody, and anti-nuclear antibody. The diagnosis of “definite SS” was made when all 4 criteria are met [27]. The exclusion criteria was as follows: all eyes diagnosed with meibomian gland dysfunction (MGD) based on the Japanese diagnostic criteria for MGD [28], eyes with punctal occlusion, subjects who wore contact lenses, subjects with iritis and severe conjunctivochalasis, subjects in whom examination of the tear meniscus could not be performed, and subjects with clinically significant cardiopulmonary, renal, or gastrointestinal tract disease, epilepsy, Parkinsonism or Parkinson’s syndrome, hypersensitivity to pilocarpine use, or who were pregnant. All subjects included in the study were instructed to not use any eye drops for at least 1 h prior to the examination in order to avoid any effect resulting from the instillation of the drops.

### 2.2. Ocular and Oral Dryness Assessed by Visual Analogue Scale

Ocular and oral dryness were evaluated by use of a 100 mm visual analogue scale (VAS) (0: no symptoms; 100 mm: maximum symptoms).

### 2.3. Evaluation of Tear Volume Changes by Meniscometry

To evaluate changes in tear volume, a video meniscometer was used to measure the R [23,24] of each eye before the administration of oral 5 mg pilocarpine (baseline R), and at 15 (R:(15)), 30 (R:(30)), and 60 (R:(60)) minutes after the administration. The change in R (ΔR = R:(15)—baseline R, mm) was then calculated.

Briefly, in this examination, a rotatable projection system with a target comprising a series of black and white stripes (four black stripes and five white stripes, each 4 mm wide) was introduced coaxially using a half-silvered mirror. The coaxial alignment of the video-meniscometer permits the meniscus of either eye to be accessed readily and allows the real-time recording of meniscus behavior over a 1·1 × 1·5 mm^2^ rectangular area of the meniscus. Images recorded with a digital video recorder were transferred to a computer, and image analysis software was used to calculate the radius of the curvature of the meniscus via the application of the concave mirror formula.

### 2.4. Examination of the Tear Film Lipid Layer

The spread grade (SG) of the tear film (TF) lipid layer (TFLL) (SG: 1–5: 1 being the best) was evaluated using the low-magnification-mode rectangular area (6.8 mm (vertical) × 8.8 mm (horizontal)) of a video interferometer (DR-1; Kowa Company, Ltd., Nagoya, Japan) [29,30]. Briefly, by illuminating the TFLL with a white light source, light interference images from the TFLL were observed. In normal eyes, the upward spread of the TFLL ceases within 2 s after the eye is opened and the velocity of the spread decreases depending on the amount of aqueous tear deficiency [31]. The behavior of the TFLL spread can be evaluated based on the patterns of the spread being classified into one of the following five grades: Grade 1—quick and complete spread; Grade 2—slow and complete spread; Grade 3—slow and partial spread (i.e., >1/2 of the observed area); Grade 4—slow and partial spread (i.e., ≤1/2 of the observed area); or Grade 5—no observed spread. The grading system used in this study was a modified version of the method previously described by Yokoi and associates, as there reportedly is a significant relationship between SG and total tear volume over the ocular surface and the decrease of the tear volume is noted as the grade increases [32].

### 2.5. Ocular Surface Examinations

The measurement of fluorescein BUT (FBUT) and the scoring of the ocular surface staining was performed using a slit-lamp microscope with a cobalt blue filter and blue-free filter [33] after the staining of tears with sodium fluorescein. After 2 drops of saline solution were instilled onto a fluorescein test strip (Ayumi Pharmaceutical Co., Tokyo, Japan), the strip was vigorously shaken and then gently touched to the margin of the central lower eyelid to stain the ocular surface with fluorescein, which was then followed by several natural blinks. Subsequently, the FBUT was counted as the time (in seconds) until the first appearance of a dark spot in the precorneal TF when the eye was kept open. FBUT was measured 3 times, and then averaged, and then evaluated before and at 60 min after the administration of oral 5 mg pilocarpine.

Corneal epithelial damage (CED) was evaluated in accordance with Miyata’s grading system (i.e., the area of superficial punctate keratopathy was graded from A0 through A3, and the density was graded from D0 through D3) [34], following the FBUT measurements and prior to the administration of oral 5 mg pilocarpine.

The Schirmer 1 test without topical anesthesia was performed using a standard Schirmer test strip (Ayumi Pharmaceutical Co.), following the FBUT measurement at 60 min after oral 5 mg pilocarpine administration. Briefly, the strip was placed for 5 min at the temporal one-third of the lower conjunctival fornix of the eye, and the length (in mm) of the filter paper that had been wetted was then recorded.

### 2.6. Evaluation of Side Effects

The side effects of sweating, headache, nausea, and diarrhea were evaluated at 60 min after oral 5 mg pilocarpine administration, and the patients were asked to grade any side effects as mild, moderate, or severe.

### 2.7. Comparison between PSS and SSS

The Schirmer 1 test, FBUT (at baseline and at 60 min after administration), R (at baseline and at 15, 30, and 60 min after administration), and both ocular and oral dryness VAS score (at baseline and at 60 min after administration) were compared between PSS and SSS.

### 2.8. Statistical Analysis

Statistical analyses were performed using JMP version 11.0 software (SAS Institute, Inc., Cary, NC, USA). All results were expressed as mean ± SD. Paired *t*-tests were used for statistical comparisons of R, the FBUT, and the VAS scores. Wilcoxon’s signed-rank test was used for statistical comparison of the SG. Moreover, the correlation between the ST-1 and ΔR was evaluated, and Spearman’s rank correlation coefficients were used for the evaluation. Unpaired *t*-tests were used for statistical comparisons of the Schirmer 1 test, the FBUT, R, and the VAS scores between PSS and SSS.

## 3. Results

### 3.1. Patient Background

The patient background is shown in Table 1. FBUT ranged from 0 to 3, and 12 (22.6%) of the 53 eyes showed the instantaneous breakup of the TF simultaneously with the opening of the eye (FBUT; 0 s). CED (area) ranged from 1–2, and CED (density) ranged from 1–3. The Schirmer 1 test value ranged from 0 to 35 mm, and 36 eyes (68.0%) showed a value of ≤5  mm/5  min. Of the 27 SS cases in this study, 14 were primary SS, and the secondary SS (SSS) cases included 7 cases of rheumatoid arthritis, 2 cases of scleroderma, 1 case of Hashimoto’s disease, and 1 case of systemic lupus erythematosus.

### 3.2. Subjective Symptoms

**Figure 1:**
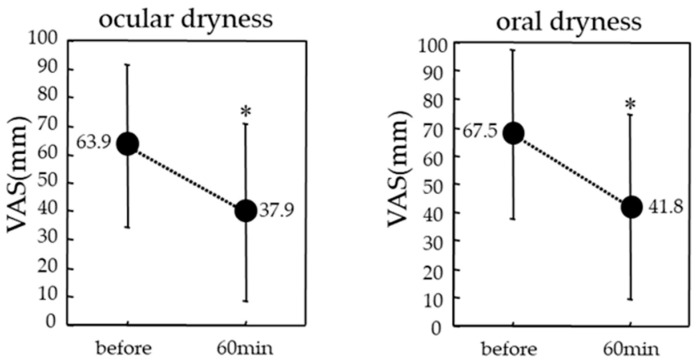
Mean visual analog scale (VAS) scores for the ocular dryness and oral dryness. Data are expressed as mean ± SD (* *p* < 0.001, paired *t*-test). A *p*-value of <0.05 is considered statistically significant.

Compared to before the administration of oral 5 mg pilocarpine, both ocular and oral dryness were found to have significantly improved at 60 min after administration, i.e., 63.9 ± 26.7 and 67.5 ± 29.9, respectively, before administration and 37.9 ± 30.3 and 41 ± 32.7, respectively, 60 min after administration (both: *p* < 0.0001) (Figure 1).

**Figure 2:**
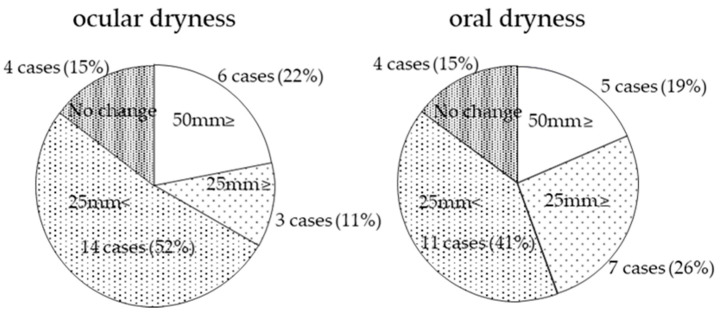
Charts illustrating the improvements of ocular dryness and oral dryness. Note: Due to rounding, percentages may not sum to 100%.

In regard to the improvement of VAS, ocular dryness improved by 50 mm or more (marked improvement) in 6 cases (22%), by 25 mm or more (moderate improvement) in 3 cases (11%), by up to 25 mm in 14 cases (52%), and remained unchanged in 4 cases (15%), thus illustrating an improvement in 85% of all cases. For oral dryness, 5 cases (19%) showed an improvement of 50 mm or more (marked improvement), 7 cases (26%) improved by 25 mm or more (moderate improvement), 11 cases (41%) improved by up to 25 mm, and 4 cases (15%) remained unchanged, thus illustrating improvement in 85% of the total cases (Figure 2).

### 3.3. Tear Volume Change

**Figure 3:**
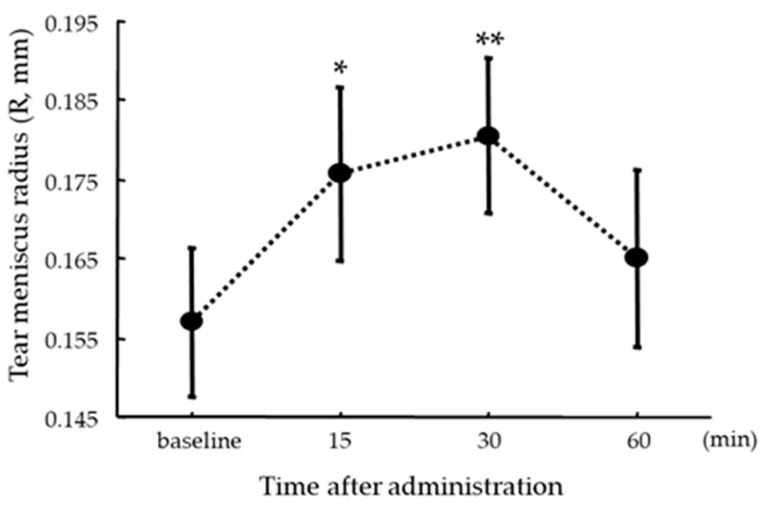
Time-dependent changes in the radius of curvature of the tear meniscus after administration of single-dose oral pilocarpine. Each datapoint represents the mean ± standard error of the mean (* *p* = 0.0313, ** *p* = 0.0025, paired *t*-test).

**Figure 4:**
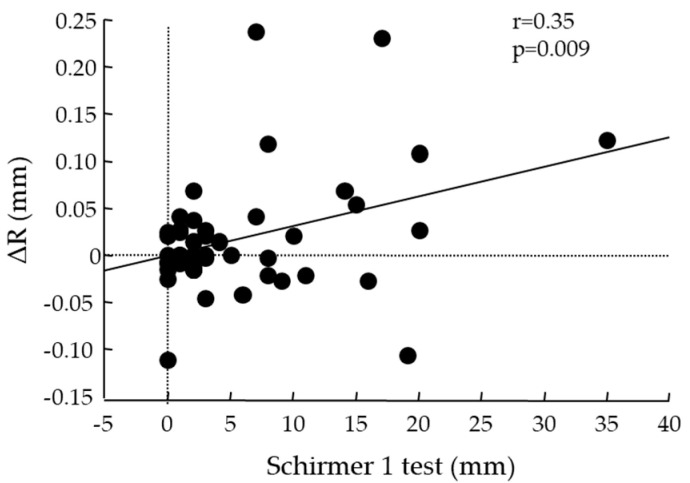
Correlation between Schirmer 1 test and ΔR. r: Spearman’s correlation coefficient; R: radius of the lower central tear meniscus curvature.

The respective R values (mean ± SD) were baseline: 0.16 ± 0.07; R:(5); 0.18 ± 0.08; R: (30): 0.18 ± 0.07; R:(60): 0.16 ± 0.08. There were significant differences between R baseline and R:(15) (*p* = 0.0313) and R:(30) (*p* = 0.0025) (Figure 3). A significant correlation was found between ST-1 values and ΔR (r = 0.35, *p* = 0.009) (Figure 4).

**Figure 5:**
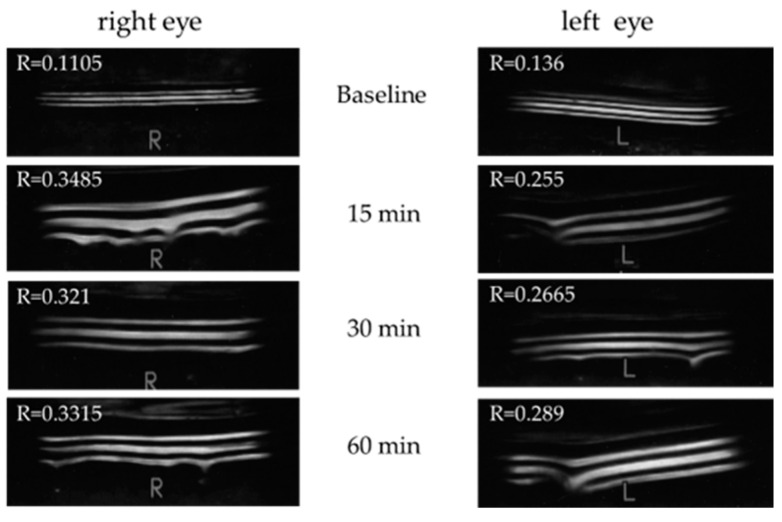
Representative images of the central lower tear meniscus of a 49–year–old female patient (Schirmer 1 test: R 7 mm, L 8 mm; fluorescein score: R A2D2, L A2D2) obtained via meniscometry at baseline and at 15, 30, and 60 min after the administration of oral 5 mg pilocarpine.

Representative meniscometry images taken of the central lower tear meniscus at baseline and at 15, 30, and 60 min after the administration of oral pilocarpine are shown in Figure 5.

### 3.4. Comparison of the SG before and at 60 min after Administration of Oral 5 mg Pilocarpine

The mean SG before and at 60 min after administration of oral 5 mg pilocarpine was 3.4 ± 1.1 (mean ± SD) and 3.3 ± 1.1, respectively (*p* = 0.63), thus illustrating that there was no significant difference between the two timepoints.

### 3.5. Comparison of FBUT before and at 60 min after Administration of Oral 5 mg Pilocarpine

**Figure 6:**
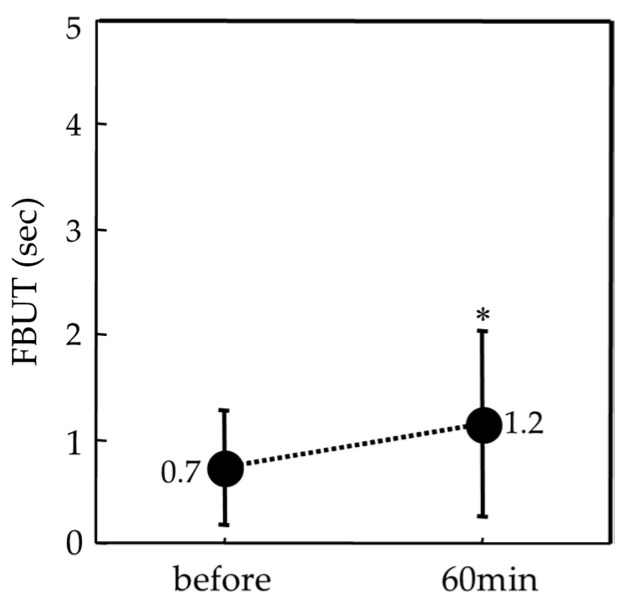
Graph showing the mean change of FBUT (in seconds) between before and at 60 min after administration of oral 5 mg pilocarpine. Data are expressed as mean ± SD (* *p* = 0.0002, paired *t*-test).

The mean FBUT was 0.7 ± 0.5 (seconds) before and 1.2 ± 0.8 at 60 min after oral 5 mg pilocarpine administration and was significantly prolonged after 60 min (*p* = 0.0002) (Figure 6).

### 3.6. Comparison between PSS and SSS

The comparison between PSS and SSS is shown in Table 2. Between the PSS and SSS, significant differences were found in FBUT at baseline (0.6 ± 0.5 and 0.9 ± 0.6, respectively; *p* = 0.04), R at 30 min (0.16 ± 0.07 and 0.20 ± 0.07, respectively; *p* = 0.02), and ΔVAS (oral dryness) (−18.8 ± 21.5 and −33.6 ± 29.5, respectively, *p* = 0.05) (Table 2).

### 3.7. Side Effects

Of the 27 cases, mild sweating was observed in 2 (7%) cases and mild nausea was observed in 1 (4%) case, and no other serious adverse reactions were observed.

## 4. Discussion

The Schirmer 1 test has long been used in the diagnosis of dry eye, and is also used in the classification criteria for SS alongside an abnormal ocular staining score [2]. Moreover, the test is used to evaluate both the nerve pathway from the trigeminal nerve to the lacrimal nerve, as well as the function of the lacrimal gland [35]. However, since it is a test of reflexing tearing, it is not suitable for the evaluation of steady-state tear volume reflecting basic tear secretion.

The tear volume at the tear menisci reportedly contains 75% to 90% of the tear fluid on the entire ocular surface [36]. In cases in which the tear meniscus can accurately be assessed, it can serve as an indicator for estimating the amount of tear volume on the ocular surface. The analysis of slit-lamp biomicroscopy images of the lower tear meniscus elucidates the “height” and “radius of curvature”, which are the best screening indicators for dry eye [37]. However, this invasive method involves the use of fluorescein instillation, and the possibility of reflective tearing cannot be ruled out. Reflective meniscometry, which projects white light onto the inferior tear meniscus, is minimally invasive and allows for measurements to be obtained under spontaneous blinking without reflective tearing [23,24].

In this present study, video-meniscometry was used to evaluate the changes in tear volume before and after the oral administration of pilocarpine hydrochloride in SS patients. After a single-dose administration of pilocarpine hydrochloride, R, which reflects not only the tear volume at the lower tear meniscus but also that over the ocular surface, increased significantly at 15 and 30 min after the administration compared to that before administration. In a previous study, we evaluated the turnover of several different ophthalmic solutions and reported that R increased significantly up to 2 min with artificial tears, 5 min with sodium hyaluronate [23], 30 min with diquafosol sodium ophthalmic solution in the normal subject group [25] after instillation, and diquafosol sodium ophthalmic solution also increased R significantly at 15 min in SS patients after instillation [26]. In the present study, the R was higher than baseline even at 60 min after pilocarpine hydrochloride administration, although the difference was not significant, thus suggesting that the replenishment of the tears is possible for up to 1 h. Furthermore, one of the advantages of the oral administration of pilocarpine hydrochloride is that it replenishes physiological tear fluid, including lacrimal gland-derived proteins such as epidermal growth factor [38] and antimicrobial proteins like lactoferrin [39] and lipocalin [40], whereas diquafosol sodium ophthalmic solution increases aqueous fluid secretion [25,41,42] and mucin secretion [43,44]. FBUT was also significantly prolonged at 60 min after administration compared to before administration, probably due to increased tear volume with the pilocarpine hydrochloride administration. We also evaluated SG, which reflects total tear volume over the ocular surface, as better spread of the TFLL can occur in cases with greater tear volume [32]. Although there was no significant difference in SG between before and 60 min after the administration of oral 5 mg pilocarpine, and this is consistent with the result that R, which had significantly increased up to 30 min after administration, decreased at 60 min and was no longer significantly different.

In this study, a correlation between the Schirmer 1 test value and ΔR was found. In patients with low Schirmer 1 test values and a severe loss of lacrimal gland function, there were few cases in which an increase in R was found, thus suggesting that oral pilocarpine hydrochloride administration is not effective in cases with a severe loss of lacrimal gland function. Hence, a good indication for pilocarpine hydrochloride administration would be cases with a Schirmer 1 test value of 5 mm or more, in which there is some degree of preserved lacrimal gland function. Sakamoto et al. [45] reported that salivary secretion was maintained after 8 years with the long-term oral administration of M3R agonists, and multivariate analysis showed a significant rate of increase in salivary secretion in patients with preserved salivary function or without severe tissue abnormalities at the initial visit. Those findings suggest that when lacrimal and salivary gland function is preserved, the early initiation and continuation of oral M3R agonists such as pilocarpine is important to maintain quality of life in patients with SS.

There are not many reports comparing dry eyes between PSS and SSS cases, although the findings in our previous reports showed no differences in Schirmer 1 test values, BUT, and serum autoantibodies between SS and PSS [46]. Other results are also inconsistent, with some reports showing that SSS with RA is less symptomatic and subclinical [47], and SSS with scleroderma had less efficacy of autologous serum eye drops compared with PSS due to elevated serum proinflammatory cytokine levels [48]. In this present study, although there was no significant difference between PSS and SSS in the Schirmer 1 test values, the R at 30 min of SSS was significantly higher than that of PSS, thus suggesting that meniscometry may be able to detect differences in lacrimal gland function which cannot be detected by Schirmer 1 test. Moreover, VAS showed greater improvement in ocular and oral dryness in SSS compared to PSS. These findings suggest that lacrimal gland function may be preserved in SSS compared to PSS; however, further studies with larger sample sizes are needed in the future.

In this study, the incidence of adverse reactions from oral pilocarpine hydrochloride was very low, i.e., two cases (7%) of sweating and one case (4%) of nausea. Eccrine sweat glands, which are sympathetically innervated but exceptionally cholinergic, may produce sweating as a pharmacological effect of pilocarpine hydrochloride; i.e., the incidence of sweating at 5 mg 3 times/day was 29% in a study by Rieke et al. [49], whereas Japanese patients reportedly had a higher incidence of sweating (i.e., 62%) at the same dose [50], probably due to physical differences. The incidence of adverse reactions is expected to increase with increasing doses, but in this study, the number of cases in which adverse reactions occurred was very low because of the single-dose administration. In clinical use, it may be necessary to start with a single dose, and then gradually increase the dose as stated in the guidelines [51].

The goal of the treatment of SS is to alleviate the exocrinopathy symptoms, as well as to control the extraglandular manifestations of the disease. Although the eye drops and cholinergic medications presented in this study are recommended in the current treatment guidelines [51], new approaches to the treatment of SS have been explored in recent years. The possibility of treating autoimmune diseases by vitamin D and probiotic supplementation has been suggested, since vitamin D deficiency and the microbiome are interrelated and are involved in autoimmunity [52,53]. Moreover, Murdaca et al. [54] reported a possible role for the IL-33/IL-31 axis in autoimmune diseases, where both cytokines cooperate in synergistic biological mechanisms in disease onset and progression, suggesting that these cytokines could be potential therapeutic targets.

It should be noted that this study had two major limitations that could be addressed in future research. First, only one male patient was evaluated in this study, and this sex imbalance could possibly have biased the results. However, SS is a female-dominated disease, and it is difficult to enroll equal numbers of male and female SS patients, especially among Asian populations due to the high ratio of female to male patients [4,55,56,57,58]. Thus, further investigation may be necessary. Second, the results in this study were obtained only after a single-dose administration. Hence, future studies involving a long-term administration over a period of several months are needed.

## 5. Conclusions

In this study, single-dose administration of oral 5 mg pilocarpine improved both oral and ocular dryness symptoms in more than 85% of the patients and increased tear fluid retention, thus suggesting that oral pilocarpine hydrochloride may be a useful treatment option for dry eye associated with SS with dry mouth.

## Figures and Tables

**Table 1:** Demographics and clinical characteristics of study subjects.

	SS (*n* = 27)
Mean Age (years)	59.2 ± 12.5
Male:Female	1:26
FBUT (seconds)	0.7 ± 0.5
CED (area)	1.5 ± 0.5
CED (density)	1.8 ± 0.5
PSS:SSS	14:13
Schirmer 1 test (mm)	5.6 ± 7.0

FBUT: fluorescein break-up time; CED: corneal epithelial damage; PSS: primary Sjögren’s syndrome; SSS: secondary Sjögren’s syndrome; Data were expressed as mean ± SD.

**Table 2:** Comparison between primary and secondary Sjögren’s syndrome.

		PSS	SSS	*p* Value
Schirmer 1 test (mm)		4.9 ± 6.2	6.4 ± 7.1	0.43
FBUT (s)	baseline	0.6 ± 0.5	0.9 ± 0.6	0.04
	60 min	1.2 ± 1.0	1.2 ± 0.5	0.97
R (mm)	baseline	0.15 ± 0.06	0.17 ± 0.08	0.26
	15 min	0.16 ± 0.07	0.20 ± 0.08	0.08
	30 min	0.16 ± 0.07	0.20 ± 0.07	0.02
	60 min	0.15 ± 0.07	0.19 ± 0.09	0.08
VAS (ocular dryness)	baseline	63.9 ± 28.3	63.8 ± 25.3	0.99
	60 min	44.5 ± 29.9	30.6 ± 29.6	0.09
VAS (oral dryness)	baseline	64.4 ± 33.2	71.1 ± 25.8	0.42
	60 min	45.6 ± 32.6	37.5 ± 32.8	0.38
ΔVAS (ocular dryness)		−19.4 ± 20.6	−33.2± 32.8	0.07
ΔVAS (oral dryness)		−18.8 ± 21.5	−33.6 ± 29.5	0.05

FBUT: fluorescein break-up time; R: radius of the lower central tear meniscus curvature; VAS: visual analogue scale; PSS: primary Sjögren’s syndrome; SSS: secondary Sjögren’s syndrome; ΔVAS = VAS (60 min)—VAS (baseline). Data are expressed as mean ± SD.

## Data Availability

The data that support the findings of this study are available from the corresponding author upon reasonable request.

## Abbreviations

BUT: breakup time; CED: corneal epithelial damage; FBUT: fluorescein BUT; M3R: M3 muscarinic acetylcholine receptor; MGD: meibomian gland dysfunction; R: radius of the lower central tear meniscus curvature; SD: standard deviation; SS: Sjögren’s syndrome; SSS: secondary Sjögren’s syndrome; VAS: visual analogue scale.
